# A Rare Case of Pellagra in a Chronic Alcoholic

**DOI:** 10.7759/cureus.47909

**Published:** 2023-10-29

**Authors:** Yasaman Navari, AmirBehzad Bagheri, Jami Foreback

**Affiliations:** 1 Internal Medicine, Michigan State University, Flint, USA; 2 Pulmonary and Critical Care Medicine, University of Michigan, Ann Arbor, USA; 3 Internal Medicine, Michigan State University at Hurley Medical Center, Flint, USA

**Keywords:** diarrhea, pruritic rash, niacin deficiency, chronic alcoholism, pellagra

## Abstract

This case report documents a rare occurrence of pellagra in a chronic alcoholic individual, characterized by a pruritic rash and gastrointestinal symptoms. The patient, a Caucasian male in his 60s, with a history of alcohol use disorder, presented with worsening skin lesions and non-bloody diarrhea. Laboratory findings revealed significant deficiencies in niacin and related metabolites, confirming the diagnosis. Prompt initiation of niacin supplementation, dietary adjustments, and supportive care led to notable improvements. This case shows the critical importance of recognizing pellagra in chronic alcoholism, emphasizing the triad of symptoms - rash, diarrhea, and malnutrition - as key diagnostic markers. Early intervention holds the potential to significantly enhance the patient's well-being and prevent disease progression.

## Introduction

Pellagra, primarily attributed to inadequate niacin (Vitamin B3) intake or absorption, traces its origins back to societies grappling with limited dietary diversity throughout history. With the passage of time, it has become intrinsically linked with chronic alcoholism, an extensively documented risk factor [1]. People with alcohol abuse may face two problems: they don't eat well and their bodies struggle to obtain the nutrients they need. The primary purpose of examining this distinctive scenario is to underscore the pivotal importance of identifying and rectifying nutritional deficiencies among those who rely on alcohol [2,3]. This case also illuminates the intricate interplay between health conditions and lifestyle choices. It inherently urges healthcare practitioners to embrace a comprehensive approach to patient well-being.

## Case presentation

A 62-year-old Caucasian gentleman, with a pertinent medical history including essential hypertension, chronic obstructive pulmonary disease (COPD), alcohol use disorder, and a long-standing history of smoking, presented to the emergency department (ED) reporting a pervasive pruritic rash and persistent diarrhea. He had been having a sedentary lifestyle and barely left his house. The onset of the rash occurred approximately two months ago and has exhibited a gradual escalation in severity. Around one month after the rash first emerged, the patient also began experiencing non-bloody diarrhea.

Upon arrival at the ED, the patient displayed evident cachexia although he remained alert and oriented. His vital signs were unremarkable. Notable findings from laboratory investigations included a diagnosis of macrocytic anemia, indicating anemia characterized by enlarged red blood cells, along with folate deficiency. Furthermore, the patient presented with hypocalcemia, hypokalemia, and mild hyponatremia, indicating imbalances in calcium, potassium, and sodium levels respectively.

**Figure 1:**
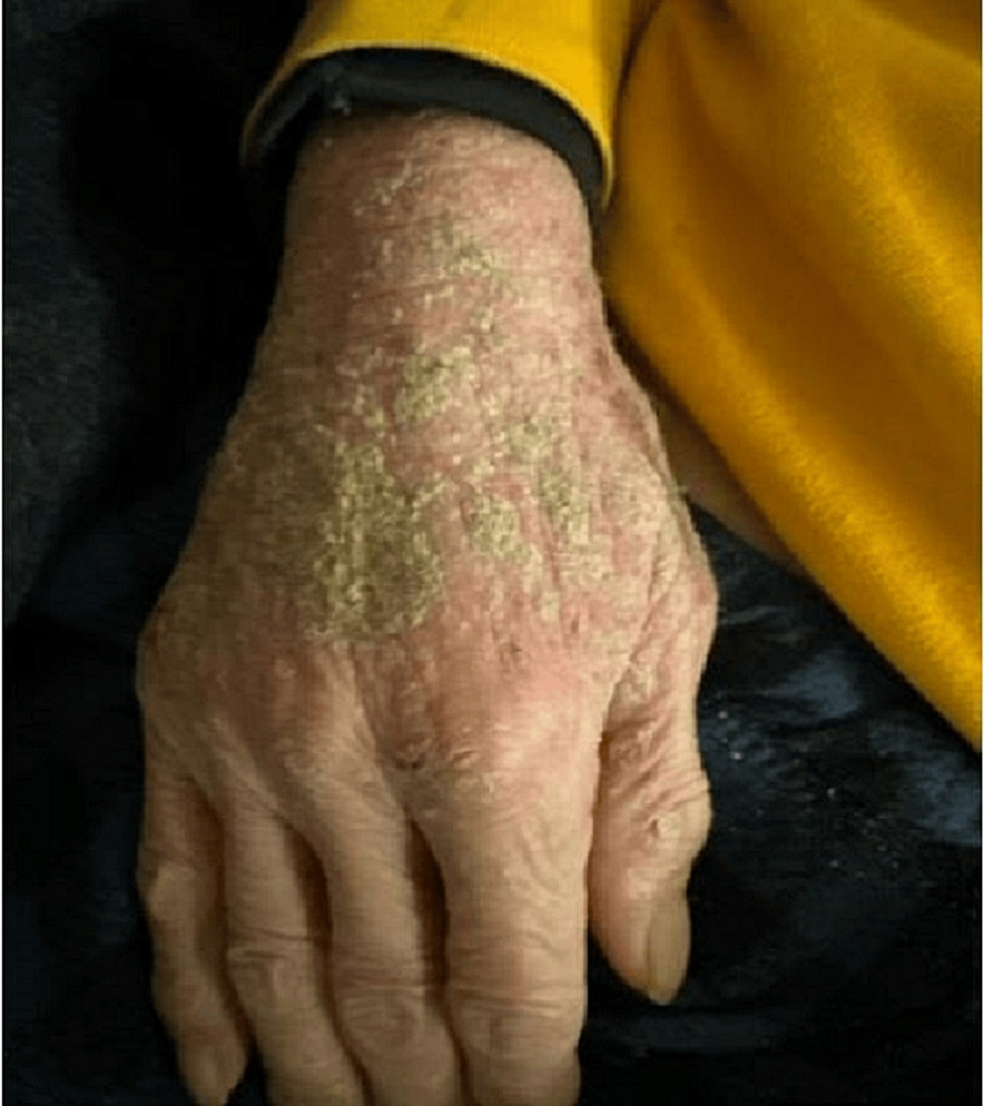
Red and brown hyperpigmentation with scaly and crusty areas in sun-exposed parts of the skin

During the physical examination, distinct erythematous, scaly, and hyperpigmented lesions were identified on sun-exposed regions of the skin (Figure 1).

Despite a prior attempt at treatment with triamcinolone cream based on an initial diagnosis of atopic dermatitis, the intervention yielded no improvement in the patient's condition. This case presentation underlines the complexity of the patient's clinical scenario, where a range of symptoms, including dermatological manifestations and gastrointestinal disturbances, requires careful evaluation and consideration for accurate diagnosis and management.

Given the patient's evident malnourished state, comprehensive evaluations were undertaken, including HIV and hepatitis panels, which yielded negative results. However, the confluence of chronic diarrhea, malnutrition, and a rash on sun-exposed areas heightened suspicions of pellagra. Lab analyses confirmed significantly diminished levels of niacin and metabolites in the patient's bloodstream, conclusively diagnosing pellagra. Importantly, other relevant diagnostic parameters remained within the normal range, further highlighting niacin deficiency as the principal causative factor.

Upon the confirmation of pellagra, a comprehensive treatment approach was initiated to address both the immediate symptoms and underlying causes. Niacin supplementation was introduced to restore the depleted nutrient levels, accompanied by a balanced diet to support the patient's recovery and overall health. Concurrently, supportive care strategies were employed to alleviate gastrointestinal discomfort. Recognizing the intricate link between alcohol dependence and nutritional deficiency, alcohol cessation counseling was incorporated into the management plan, highlighting the significance of holistic patient care.

## Discussion

The rate of pellagra is lower than 1% in the US, and the main reason is alcohol abuse [4]. This case highlights the importance of recognizing pellagra as a potential consequence of chronic alcoholism [1,2]. The combination of a characteristic skin rash, gastrointestinal symptoms, and low niacin levels aided in establishing the diagnosis promptly [5,6]. Early detection and appropriate intervention can lead to favorable outcomes and prevent potential complications associated with pellagra. The distinct triad of symptoms - rash, diarrhea, and malnutrition - served as a diagnostic hallmark, guiding further evaluation and ultimately confirming the diagnosis of pellagra through niacin and metabolite assessment. Additionally, this case report underscores the need for healthcare practitioners to understand that medical conditions are intimately intertwined with lifestyle factors.

## Conclusions

This case shows the interplay between health conditions and lifestyle choices. The unusual presentation of pellagra in a chronic alcoholic underlines the lasting impact of both physiological and behavioral factors on health outcomes. Multidisciplinary collaboration is needed with specialists from dermatology, gastroenterology, internal medicine, nutrition, and addiction counseling working together to ensure a comprehensive approach to care. By acknowledging the interconnectedness of these disciplines, effective treatment strategies were tailored to address both immediate symptoms and underlying causes. By integrating targeted interventions and collaborative care, the healthcare community can provide comprehensive, patient-centered solutions that optimize both immediate relief and long-term well-being.
